# Pragmatic Treatment of Patients With Systemic Lupus Erythematosus With Rituximab: Long‐Term Effects on Serum Immunoglobulins

**DOI:** 10.1002/acr.22993

**Published:** 2017-04-24

**Authors:** Venkat Reddy, Lina Martinez, David A. Isenberg, Maria J. Leandro, Geraldine Cambridge

**Affiliations:** ^1^ University College London, Rayne InstituteLondonUK; ^2^ Hospital General Universitario, Gregorio MarañónMadridSpain

## Abstract

**Objective:**

B cell–depletion therapy based on rituximab is a therapeutic option for refractory disease in patients with systemic lupus erythematosus (SLE). The aim of this observational study was to document long‐term effects on B cell function by following serum immunoglobulin levels in patients with SLE treated with rituximab in routine clinical practice.

**Methods:**

We included 57 consecutive patients with SLE treated with rituximab and concomitant/sequential immunosuppressants and measured serum total IgG, IgM, and IgA and IgG anti‐dsDNA antibodies, over a median of 48 months most recent followup. Flow cytometry was used prospectively to assess B cell phenotypes in 17 of 57 patients.

**Results:**

Twelve patients (21%) had persistent IgM hypogammaglobulinemia (<0.4 gm/liter), and 4 of 57 (5%) had low IgG (<7 gm/liter) at the most recent followup (range 12–144 months). This was not associated with serious adverse events or high anti–double‐stranded DNA (anti‐dsDNA) antibodies (>1,000 IU/ml; normal <50 IU/ml). Factors predictive of low serum IgM included baseline serum IgM ≤0.8 gm/liter (receiver operator curve analysis) and subsequent therapy with mycophenolate mofetil (MMF; odds ratio 6.8, compared with other immunosuppressants). In patients maintaining normal IgM levels (9 of 17), the frequency of circulating IgD+CD27+ B cells was significantly higher (*P* = 0.05). At 12 months after rituximab, 7 of 30 SLE patients with baseline anti‐dsDNA ≤1,000 IU/ml had lost seropositivity.

**Conclusion:**

Lower baseline serum IgM levels and sequential therapy with MMF were predictive of IgM hypogammaglobulinemia after rituximab in SLE, but this was not associated with higher levels of anti‐dsDNA antibodies or an increased risk of infections. This provides useful directions for clinicians regarding rituximab and sequential immunosuppressive treatment for patients with SLE.

## INTRODUCTION

Hypogammaglobulinemia can be an important adverse outcome of B cell–depletion therapy with rituximab (a chimeric anti‐CD20 monoclonal antibody). Whereas transient hypogammaglobulinemia may not require specific therapy, some patients with B cell malignancies and autoimmune diseases 1 develop persistent hypogammaglobulinemia following rituximab, requiring intravenous immunoglobulin replacement therapy, particularly in the context of recurrent infections 2, 3, 4. Although antimicrobial antibody responses are relatively robust, the degree of response to challenge with influenza, pneumococcal, and tetanus immunogens after rituximab treatment may be impaired. This appears to relate to the degree and duration of B cell depletion in peripheral blood in patients with rheumatoid arthritis (RA) and systemic lupus erythematosus (SLE) 5, 6, 7.

Box 1 **Significance & Innovations**

- IgG hypogammaglobulinemia was rare in systemic lupus erythematosus (SLE) patients at long‐term followup after multiple cycles of rituximab (RTX).
- RTX normalized raised IgG in the majority of patients.
- Low levels of serum total IgM presented in nearly one‐quarter of SLE patients and were associated with lower baseline levels, older age, and sequential therapy with mycophenolate mofetil.
- Low IgM was not associated with persistently high levels of anti–double‐stranded DNA or adverse events.

B cell–depletion therapy based on rituximab is as yet unlicensed for SLE, but is used to treat early‐onset and refractory disease 8, 9, 10, 11. However, the probability of and factors associated with the incidence of persistent hypogammaglobulinemia after rituximab in the routine clinical setting has not been explored. Therefore, it is of direct clinical relevance to identify factors that may predict those at an increased risk of developing persistent hypogammaglobulinemia.

Both underlying disease and immunosuppressive therapies may affect serum Ig levels. In patients with SLE, hypergammaglobulinemia is often present; paradoxically, however, hypogammaglobulinemia similar to common variable immunodeficiency occasionally occurs and may relate to the presence of lymphocytotoxic autoantibodies 12, 13. Selective IgM and IgA deficiency has also been reported 14, 15. Hypogammaglobulinemia may be associated with older age, low IgG at baseline, nephritis 4, and treatment with immunosuppressants, including cyclophosphamide and mycophenolate mofetil (MMF) 16, 17, 18. A higher cumulative dose or repeated cycles of rituximab and concomitant or sequential use of immunosuppressants appear to increase the risk of persistent hypogammaglobulinemia in antineutrophil cytoplasmic antibody–associated vasculitis (AAV) and other autoimmune diseases 4. Importantly, long‐term persistence of hypogammaglobulinemia and associated adverse events is better appreciated in long‐term followup studies than short‐term clinical trials. Such information could therefore serve to identify those at a higher risk.

B cell hyperactivity, characteristic of SLE, results in excessive production of both polyclonal and autoreactive antibodies 19, even before the onset of clinical disease 20, 21, 22. Elevated levels of IgG anti–double‐stranded DNA (anti‐dsDNA) antibodies are characteristic of SLE and considered pathogenic, but may occur independently of hypergammaglobulinemia 23, 24. Immune dysregulation in patients with SLE is at least partly related to changes in the interactions between immune cells within germinal centers and altered trafficking of peripheral blood lymphocytes 25, 26. Some abnormalities in the composition of peripheral B cell phenotypes appear to “improve” following rituximab, but this may also reflect naive B cell return, which recapitulates ontogeny 27, 28. Reduced levels of possibly protective natural antibodies of the IgM class have been suggested to be associated with development of anti‐dsDNA antibodies in a murine model of SLE 29.

A study of the recovery of B cell subpopulations in patients with SLE who develop hypogammaglobulinemia may also relate to the extent of B cell depletion in the short term, and to the recovery of B cell subpopulations and/or clones in the long term, both of which may impact serum Ig levels. To this end, we investigated whether baseline serum Ig levels, concomitant/sequential immunosuppressants, and B cell phenotypes predict the development and/or persistence of hypogammaglobulinemia after rituximab. The relationship between serum Ig levels and anti‐ds DNA antibodies was also determined over the course of the study.

## PATIENTS AND METHODS

#### Patients

In this cross‐sectional observational study, 57 consecutive patients with SLE, who met the revised American College of Rheumatology classification criteria 30 and were treated with rituximab, were included. All patients were attending University College London Hospitals (UCLH) and were treated according to clinical need. The specific indication for rituximab treatment in this cohort was persistent active disease refractory to conventional immunosuppressive therapies. Clinical notes and laboratory results of all SLE patients treated with rituximab from January 2000 until December 2012 were reviewed retrospectively. As this study was a clinical evaluation, it did not require hospital ethics committee approval, and results were compiled from anonymized files. In the cross‐sectional B cell phenotype study, collection of blood samples was approved by the UCLH Ethics Committee. Patients gave written informed consent according to the Declaration of Helsinki.

All patients had active disease refractory to hydroxychloroquine and at least 2 conventional immunosuppressants, including azathioprine (AZT), MMF, methotrexate (MTX), or cyclophosphamide (CYC). A majority of patients continued with low‐dose corticosteroids (prednisolone <10 mg/day), but in most cases the use of other immunosuppressants was stopped until evidence of B cell return (CD19+ cells >5/µl) or started only as required for optimal control of disease activity. A typical rituximab treatment cycle consisted of rituximab, 2 doses of 1 gm given 1–2 weeks apart in combination with 1 dose of intravenous cyclophosphamide (750 mg). The clinical response to rituximab in this cohort has been reported previously 31. Clinical and laboratory parameters were analyzed during the first cycle of rituximab (up to 12 months) and the most recent time point from all patients, some of whom had received multiple cycles of rituximab‐based treatment in combination with concomitant and/or subsequent therapy with immunosuppressants to determine longer‐term effects on the recovery of B cell subpopulations, serum Ig, and autoantibodies.

#### Clinical and laboratory indices

Serum levels of IgG, IgM, and IgA, and IgG anti‐dsDNA antibody levels, were recorded from baseline (before the first infusion of rituximab) up to 12 months after rituximab, and also at most recent followup. Hypogammaglobulinemia was defined by serum IgG <7 gm/liter (normal range: 7–16 gm/liter), IgM <0.4 gm/liter (normal range 0.4–2.3 gm/liter); and IgA <0.7 gm/liter (normal range: 0.7–4.0 gm/liter).

#### B cell immunophenotyping

B cell phenotypes in whole blood were prospectively characterized by flow cytometry. Samples were stained with fluorescence‐tagged antibodies against CD19 (phycoerythrin [PE]–Cy7), IgD (fluorescein isothiocyanate), and CD27 (PE) (BD Biosciences). B cells were identified as CD19+ cells in the lymphocyte‐gated cells. B cell phenotypes were identified as follows: naive, IgD+CD27−, unswitched memory IgD+CD27+, switched memory IgD−CD27+, and double negative IgD−CD27−.

#### Statistical analysis

Statistical analysis was performed using Graph Pad Prism, version 5.01. Matched‐pairs signed rank test and the Mann‐Whitney U test were used to analyze differences in serum Ig between paired and unpaired data, respectively. Spearman's rank test was used to analyze correlations. Fisher's exact test was used to compare the proportions of patients in different groups. Receiver operator curve analysis was used to identify the cutoff value that distinguished patients developing low Ig. The odds ratio was used to express the effect of concomitant and/or subsequent immunosuppressants on the development of hypogammaglobulinemia.

## RESULTS

#### Patient demographics

Clinical features, drug therapies prior to RTX and at most recent followup, and serology are shown for patients with low serum IgM and for those retaining normal levels of IgM in Supplementary Tables 1 and 2, respectively (available on the *Arthritis Care & Research* web site at http://onlinelibrary.wiley.com/doi/10.1002/acr.22993/abstract). Lupus nephritis was diagnosed in 29 of 57 patients (51%). Percentages of patients in each group were similar (7 of 12 [58%] in the low IgM group and 22 of 45 [49%] in the normal IgM group). Detailed patient demographics and clinical responses of this cohort of 57 patients have been previously described 31, 32, 33. The median age at the time of the first rituximab treatment cycle was 34 years (range 17–74 years). All patients had received previous treatment with at least 2 different immunosuppressants, not including corticosteroids, which were continued at a low dose (prednisolone <10 mg/day). The median duration of followup was 48 months (range 12–144 months).

#### Serum total Ig and anti‐dsDNA antibodies in patients at 12‐month followup after rituximab

**Figure 1:**
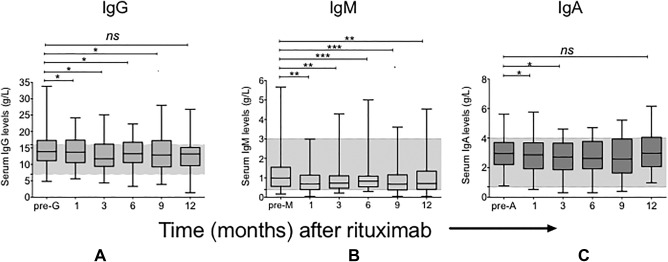
Serum levels of IgG (**A**), IgM (**B**), and IgA (**C**) at intervals up to 12 months after treatment of patients with systemic lupus erythematosus with rituximab (n = 32). Box and whiskers represent median, interquartile range, and range. Differences between baseline and subsequent immunoglobulin levels of each class were calculated using Wilcoxon's matched‐pairs signed rank test. * = *P* < 0.05; ** = *P* < 0.005; *** = *P* < 0.0005; ns = not significant.

**Figure 2:**
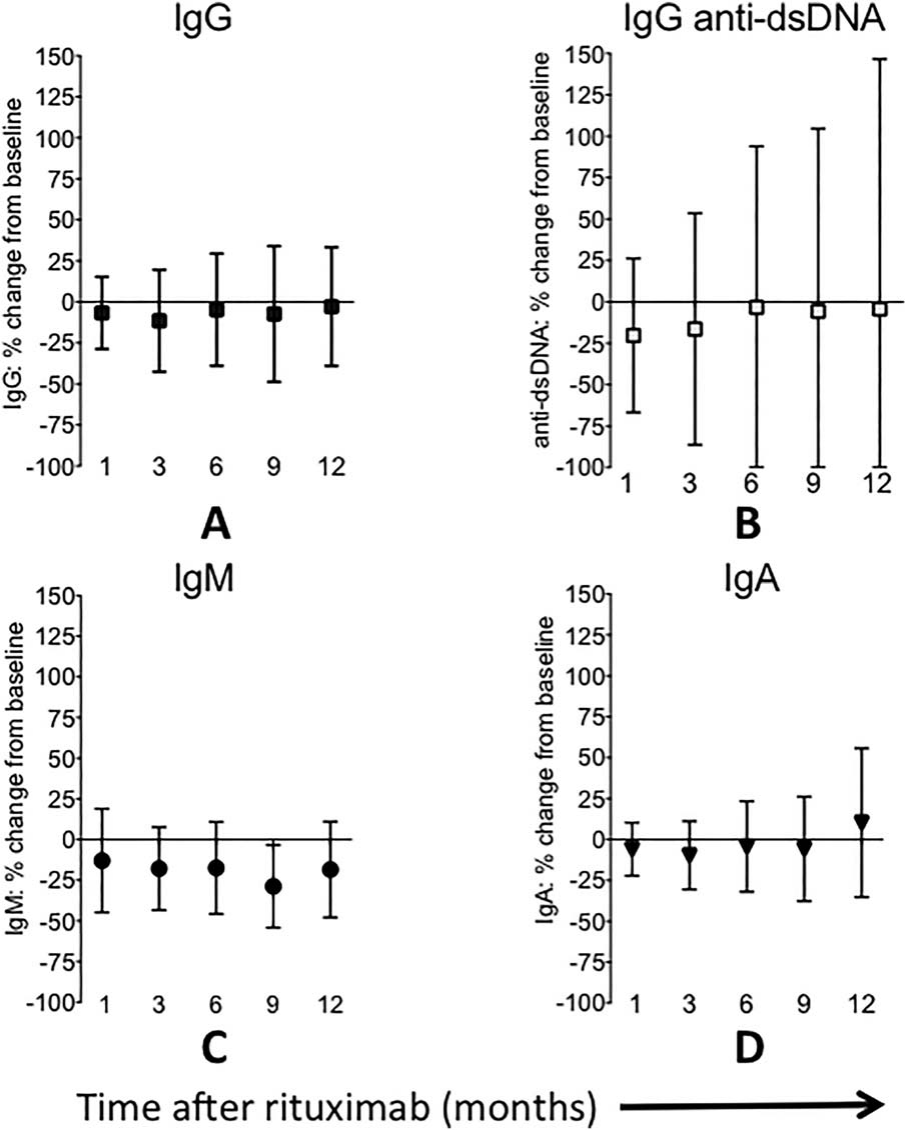
Percentage change from baseline in immunoglobulin classes and anti–double‐stranded DNA (anti‐dsDNA) antibodies. Means and SDs of serum IgG (**A**), anti‐dsDNA autoantibodies (**B**), IgM (**C**), and IgA (**D**) at baseline and up to 12‐month followup after initial cycle of rituximab (administered at time 0) are expressed as a percentage of baseline (pre‐rituximab) levels.

At baseline, 3 patients had low IgM, none had low IgA, and 1 had low IgG (Figure 1A, B, and C). Eleven patients had raised serum IgG levels (>16 gm/liter). At 12‐month followup after rituximab (n = 32), median baseline serum IgG level was 13.9 gm/liter, which was significantly reduced at 1, 2, 6, and 9 months after rituximab (*P* < 0.05 for all), but similar to baseline levels at 12 months (median 13.2 gm/liter) (Figure 1A). The median IgM levels, however, were significantly lower at all time points (Wilcoxon's matched‐pairs signed rank test; *P* < 0.005 for all), with the median serum IgM level at baseline (1.0 gm/liter) decreasing significantly to 0.71 gm/liter at 12 months (Figure 1B). The median baseline serum IgA level was 2.9 gm/liter, which fell at 1 and 3 months (*P* < 0.05) only (Figure 1C). The percentage change from baseline to 12 months of serum Ig and anti‐dsDNA is shown in Figure 2, and we noted a hierarchy in percent reduction from baseline with IgM > IgG > IgA (−18.4%, −2.8%, and 10.3%; Figure 2A, C, and D, respectively) and remarkable variations in IgG anti‐dsDNA levels (Figure 2B).

#### Serum Ig and anti‐dsDNA levels at 12 months after rituximab: relationship with baseline

##### Serum IgM

**Figure 3:**
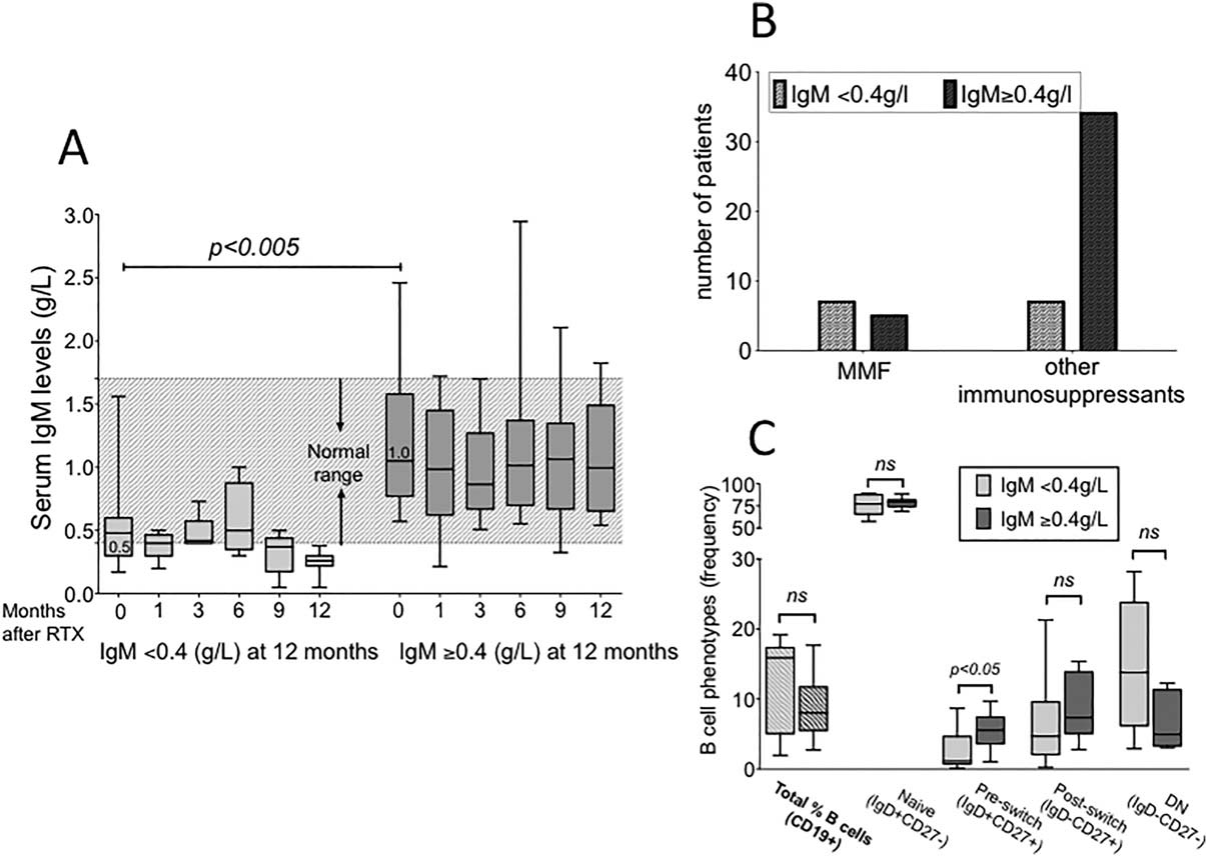
Development of IgM hypogammaglobulinemia after rituximab: relationship with baseline serum IgM, sequential therapy, and B cell phenotype. **A,** Results for serum IgM were grouped on the basis of being within the normal range (0.4–2.3 gm/liter) at 12 months (indicated by shaded area) or <0.4 gm/liter at 12 months. Box and whiskers show median, interquartile range (IQR), and range with significance between baseline values in each group, calculated using the Mann‐Whitney U test. **B,** The number of patients who developed IgM hypogammaglobulinemia (IgM <0.4) and who had been treated with mycophenolate mofetil (MMF; 7 of 12) or with other immunosuppressants (7 of 43) following rituximab (RTX) are shown (odds ratio 6.8, 95% confidence interval 1.66–27.77). **C,** The frequency (%CD19+ B cells) of B cell subpopulations in samples available from patients with low (<0.4 gm/liter; n = 8) or serum IgM levels within the normal range (n = 9) after RTX. B cell subpopulations were defined using relative expression of IgD and CD27. Box and whiskers represent the median, IQR, and range of values and significance calculated using the Mann‐Whitney U test. Significance was at 5% level. ns = not significant; DN = double negative.

At 12 months of followup, 25% of SLE patients (8 of 32) had serum IgM levels below the normal range. There was a significant difference between median baseline serum IgM levels in patients who developed low serum IgM levels at 12 months (8 of 32) and those who did not (*P* < 0.005) at 0.5 and 1.0 gm/liter, respectively (Figure 3A).

##### Serum IgG

**Figure 4:**
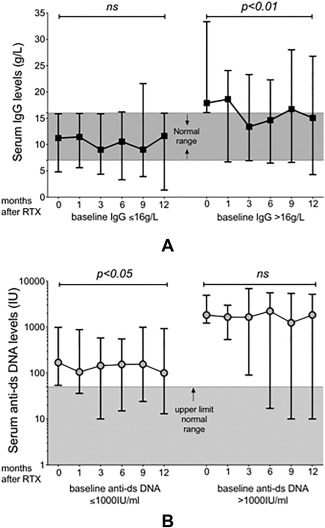
Fluctuations in serum IgG and anti–double‐stranded DNA (anti‐dsDNA) antibody levels in relation to baseline levels. **A,** Patients (n = 32) were grouped on the basis of whether their baseline serum IgG levels were within the normal range or greater than the upper limit of normal range (normal range 7–16 gm/liter; shaded area). Median and range for serum IgG levels for up to 12 months after rituximab (RTX) are shown. **B,** IgG anti‐dsDNA antibody levels in seropositive patients following RTX are shown for followup of 12 months. Upper limit of positive test was 50 IU/ml, and the shaded area indicates normal range. Results were stratified according to baseline anti‐dsDNA antibody levels of ≤1,000 IU/ml or >1,000 IU/ml. Significance between baseline in each group and 12‐month values were calculated using Wilcoxon's matched‐pairs signed rank test (significance level at 5%).

Only 1 patient had low serum IgG before treatment and at 12 months, and only 2 additional patients had levels <7 gm/liter. Figure 4A shows that in patients with IgG hypergammaglobulinemia pre‐rituximab (median 17.9 gm/liter), there was a significant reduction in median IgG levels between baseline and 12‐month followup in 11 of 32 patients (34%) (*P* < 0.01; Wilcoxon's matched‐pairs signed rank test), with levels normalizing in 8 patients. In those with baseline IgG within the normal range, there was no difference between baseline and levels at 12 months postrituximab.

##### Serum IgA

Similarly, there was a trend toward higher baseline median serum IgA levels, with a reduction in serum IgA levels when compared with baseline median serum IgA levels and with those who did not, at 3.7 gm/liter and 2.7 gm/liter (*P* = 0.06) (data not shown).

##### Anti‐dsDNA antibodies

At 12 months, in 8 of 32 patients (19%), the levels of anti‐dsDNA fell to within the normal range, but median levels at baseline and 12‐month followup were similar (169 versus 100 IU/ml, not significant) (data not shown). In Figure 4B, patients were divided on the basis of having anti‐dsDNA titers ≤ or >1,000 IU/ml. In those with anti‐dsDNA levels >1,000 IU/ml, only 1 of 9 had levels that fell to within the normal range at 12 months compared with 7 of 23 (35%) who had levels ≤1,000 IU/ml at baseline. A significant reduction in median levels after 12 months in patients with titers ≤1,000 IU/ml, but not in those with titers >1,000 IU/ml, was also noted (Figure 4B).

#### Effect of rituximab on serum Ig at most recent followup

**Figure 5:**
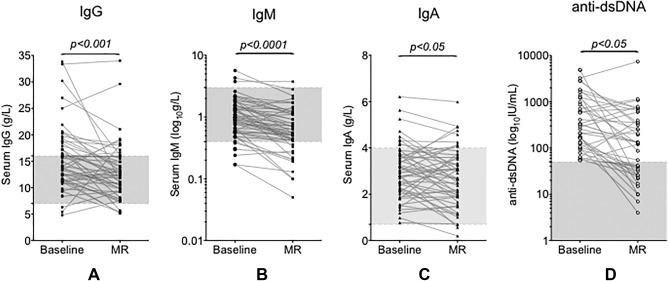
Changes between baseline serum Ig and anti–double‐stranded DNA (anti‐dsDNA) levels in systemic lupus erythematosus patients at the most recent (MR) followup. Paired serum Ig levels were available from 54 patients and anti‐dsDNA antibody levels from 40 patients. Shaded areas indicate the normal ranges used for each parameter. Values for serum levels of IgG **(A)**, IgM **(B)**, IgA **(C),** and anti‐dsDNA antibodies **(D)** at baseline and at MR followup (ranging from 12–144 months after initial rituximab treatment) are shown. Significance values shown were given by Wilcoxon's matched‐pairs signed rank tests for values at MR followup compared with baseline.

Low serum IgM was present in 12 of 57 patients (21%) (Figure 5B), with only 4 of 57 patients developing low IgG, all of whom had normal pretreatment levels (Figure 5A). Of 21 patients with raised serum IgG at pretreatment, 15 had normalized at the most recent followup. The demographics of the 12 patients developing low serum IgM levels following rituximab are shown in Supplementary Table 1 (available on the *Arthritis Care & Research* web site at http://onlinelibrary.wiley.com/doi/10.1002/acr.22993/abstract). Interestingly, at 12 months, 8 of 32 patients (25%) had low IgM, and at last followup a similar proportion, 12 of 57 (21%), had low IgM, suggesting that an accumulated rituximab dose was not necessarily related to development of low serum IgM.

There was no difference in sex distribution compared with the whole cohort (data not shown), but they tended to be older (median age 43 years, range 22–59 years) compared with those maintaining normal IgM levels (median age 32 years, range 21–74 years) (Mann‐Whitney U test, *P* < 0.01). Three of 56 patients developed low IgA levels (Figure 5C). In 15 of 40 patients (38%), anti‐dsDNA levels normalized, with the majority (13 of 15) having had levels ≤1,000 IU/ml at baseline (Figure 5D). No serious adverse events were observed 31, 33, and none of the patients required intravenous Ig therapy.

#### Relationship between low serum IgM and IgG anti‐dsDNA antibody levels

We did not find significant correlations between baseline serum IgM and anti‐dsDNA levels at baseline or maximal followup (data not shown). Further, median serum IgM levels in patients with anti‐dsDNA >1,000 IU/ml and those with levels ≤1,000 IU/ml (1.1 gm/liter and 0.9 gm/liter, respectively) were not significantly different (Mann‐Whitney U test; data not shown).

#### Predictive factors for the development of low serum IgM after rituximab

IgG hypogammaglobulinemia was present at long‐term followup in only 5% of SLE patients. As shown in Figure 3A, however, patients with serum IgM levels below 0.4 gm/liter at 12 months after rituximab had significantly lower baseline levels than those with IgM within the normal range at 1 year followup (*P* < 0.005). We employed receiver operating characteristic analysis (Supplementary Figure 1A, available on the *Arthritis Care & Research* web site at http://onlinelibrary.wiley.com/doi/10.1002/acr.22993/abstract) and found that a pretreatment serum IgM level ≤0.8 gm/liter was associated with a greater than 3‐fold likelihood ratio for the development of low IgM (<0.4 gm/liter) at 12 months, as well as at long‐term followup comparing those with low serum IgM (n = 12) and those with normal serum levels (n = 43) (Supplementary Figure 1B, available on the *Arthritis Care & Research* web site at http://onlinelibrary.wiley.com/doi/10.1002/acr.22993/abstract), with a significant area under the curve of 0.85 (95% confidence interval [95% CI] 0.7–1.0, *P =* 0.0002). This finding suggested that baseline serum IgM ≤0.8 gm/liter was associated with a greater than 4‐fold increase in the likelihood of developing low IgM (sensitivity 83%, specificity 80%). In accord with this cutoff value, at most recent followup, we found that 10 of 18 patients with serum IgM ≤0.8 gm/liter at baseline, and only 2 of 37 patients with levels >0.8 gm/liter, developed low IgM (*P* < 0.0001 by Fisher's exact test).

#### Effect of sequential therapy with immunosuppressants

The results from our extended data at most recent followup showed that 12 of 57 patients developed low IgM, and 6 of these patients (50%) were treated with MMF at least 6 months after rituximab and 2 months before the time of analysis (Supplementary Table 1, available on the *Arthritis Care & Research* web site at http://onlinelibrary.wiley.com/doi/10.1002/acr.22993/abstract). In contrast, only 7 of 43 patients (16%) treated with other immunosuppressants, including AZT, MTX, and CYC, developed low IgM (Figure 3B). The odds ratio for the analysis was 6.8 (95% CI 1.66–27.77).

#### B cell phenotypes in patients with low serum IgM levels

We found that the frequency of unswitched B cells (IgD+CD27+), but not other phenotypes, was significantly lower in patients who developed low IgM after rituximab when compared with those who did not (*P* < 0.05), although a trend for higher frequency of double negative (IgD−CD27−) memory B cell subpopulation was also apparent (n = 8) (Figure 3C). We found no significant difference between sex distribution or age, median times since last rituximab treatment (24 months in the low IgM group, 15 months in those with normal IgM), or in percentage of B cells, levels of C3, cumulative dose of rituximab, or anti‐dsDNA levels between the groups (Supplementary Tables 1 and 2, available on the *Arthritis Care & Research* web site at http://onlinelibrary.wiley.com/doi/10.1002/acr.22993/abstract) (data not shown).

## DISCUSSION

In this study, we found that lower baseline IgM levels were predictive of low serum IgM after rituximab and associated with a lower frequency of unswitched memory B cells. Sequential treatment with MMF after rituximab was also associated with low serum IgM. IgG hypogammaglobulinemia was rare, and the majority (71%) of those with raised serum IgG at baseline had normalized at maximum followup. Patients with low serum IgM did not experience serious adverse events. In the most recent published results of the cohort from which these patients were derived, we showed that the safety profile was favorable, and infusion related and hypersensitivity reactions were mostly mild to moderate 33.

There was a disparity in the dynamics of fluctuations between isotypes of serum Ig after rituximab. At 12 months and also at long‐term followup, median levels of serum IgG and IgA were not significantly different from those at baseline, with very few patients developing low levels of IgG or IgA. This contrasts with our experience in patients with RA in whom those with lower baseline serum Ig levels tended to develop persistent IgM and IgG hypergammaglobulinemia, resulting from an accumulation of incremental decreases after repeat cycles. The incidence of low IgM increased from 9.2% to 38.8% and that of IgG from 11.8% to 22.2% of RA patients, after 1 and 5 cycles, respectively 34. In patients with SLE, however, our results show that the incidences are much lower after repeat cycles, being for IgM 12 of 57 patients (21%), but only 4 of 57 patients (7%) developing low IgG, with all retaining IgG levels >5 gm, and therefore none were treated with intravenous immunoglobulin. Interestingly, at 12 months, 8 of 32 patients (25%) had low IgM, and at last followup a similar proportion, 12 of 57 patients (21%), had low IgM, suggesting that accumulated rituximab dose was not necessarily related to development of low serum IgM. An important factor influencing serum Ig levels is the balance between synthetic and catabolic rate of different Ig isotypes. IgG catabolism is greater in patients with SLE than in patients with RA, whereas IgM catabolism is greater in RA compared to patients with SLE 35.

In patients with AAV and thrombotic thrombocytopenic purpura, co‐therapies such as cyclophosphamide and plasmapheresis make it difficult to dissect the role of rituximab per se in the development of low serum Ig. None of our patients received plasmapheresis, but patients with SLE usually receive a single dose of 750 mg cyclophosphamide, which is substantially lower than that used in AAV. The comparison between patient groups was also confounded due to lower pre‐rituximab serum IgG levels in patients with AAV 4. Of direct clinical relevance, rituximab treatment did not result in significant reductions in serum IgG levels in those with low baseline IgG levels of <6 gm/liter 4. Even allowing for these limitations, we found that incidences of low IgG levels in SLE were markedly less frequent than in patients with AAV and RA.

There was no association between serum IgM levels, or with the development of low IgM, with levels of IgG anti‐dsDNA antibodies. Patients who developed low IgM however, had 2‐fold lower median levels of serum IgM before rituximab compared with those without IgM hypogammaglobulinemia at long‐term followup. Differential effects on Ig classes have also been described in patients with RA, as well as in patients with multiple myeloma treated with autologous hematopoietic stem cell transplant (HSCT) and rituximab maintenance therapy 36. Both groups of patients tend to develop low IgM, but not IgG and IgA. In contrast, some patients with refractory follicular lymphoma treated with rituximab and HSCT developed persistently low IgA and IgG with recovery of IgM levels 34, 37. Underlying disease can therefore influence the development of isotype‐specific hypogammaglobulinemia.

Serum IgM is derived from both (short‐lived) newly generated perifollicular B cells (CD27−) and from CD27+ (unswitched) marginal zone B cells 38. Differences in specific co‐therapies or the regimen used may also therefore account for some of the disparity between the isotypes affected, depending on the parent B cells affected. Serum levels of IgA and IgG are largely maintained by long‐lived (CD20−) plasma cells, predominantly in the bone marrow. These are therefore not directly targeted by rituximab, and protective immunity is largely maintained, as in RA patients, for example (34,39). It is, however, difficult to differentiate the direct effects of rituximab preventing formation of new plasma cells from indirect effects through disease control.

We found no difference between time since last rituximab infusion or in cumulative rituximab dose in the subgroup of SLE patients studied for B cell phenotype. In SLE, MMF, but not AZT or hydroxychloroquine, treatment has been associated with reduced frequency of switched memory B cells and modest decreases in levels of serum Ig and of anti‐dsDNA antibodies 40, 41, 42. The composition of B cell subpopulations may vary between individuals with SLE and after rituximab; repopulation appears to recapitulate ontogeny, perhaps further influenced by antigen stimulation 43. We found that the frequency of unswitched (IgD+CD27+) B cells was significantly lower in patients who developed low IgM after rituximab when compared with those who did not. Relative levels of immunoglobulin may relate to the composition of B cell pools in bone marrow, lymphoid, and inflammatory tissues, which differ between individual patients. This is supported by the finding that patients who developed low IgM already had lower baseline levels 44. Unswitched (IgM‐committed) B cells are preferentially depleted by rituximab in vitro, suggesting a reduced threshold for survival and slow regeneration of unswitched B cells in SLE 45, 46, 47.

Our results indicated a possible association between sequential treatment with MMF after rituximab and low serum IgM. MMF preferentially targets type II inosine monophosphate dehydrogenase, which is up‐regulated in activated lymphocytes (both B and T lymphocytes) 48, 49. Rituximab preferentially depletes naive and unswitched B cells, both of which are direct precursors for IgM production. Together with the potential removal of activated naive and memory cells by MMF, this may explain the profound effect of using a combination of rituximab and MMF on serum IgM levels. Co‐therapy with MMF has been associated with a higher rate of infections in clinical trial studies using ocrelizumab 50, and low Ig was noted in patients treated with a combination of MMF and atacicept 51. In the later study, low IgM levels in SLE patients treated with MMF alone in the placebo arm did not recover over the course of the study. Our data confirm that caution is needed when the combination of MMF, with judicious administration of the dose, and rituximab is being considered.

In contrast to IgM, serum IgA, after an initial decrease in median levels, started recovering as early as 2 months after rituximab, approaching baseline levels by 6 months. Early recovery in serum IgA levels suggests that the IgA plasma cell pool was rapidly replenished. It has previously been reported that circulating IgA+ plasmablasts can remain detectable early after rituximab, suggesting resistance to depletion of switched IgA+ precursor B cells, likely in the mucosal microenvironment and/or early regeneration 52. Serum IgG levels, despite showing a longer “lag” when compared with the recovery of serum IgA levels, was apparently also sustainable, attaining pretreatment levels in most patients by 12 months after rituximab. Indeed, rituximab treatment resulted in correction of hypergammaglobulinemia in most patients in our cohort. At long‐term followup, very few (5% of patients) had serum IgG levels below the lower limit of the normal range.

Percentage change from baseline of dsDNA antibodies was highly variable between patients. Differences in patterns of fluctuations in anti‐dsDNA antibodies between patients implied a variable contribution from anti‐dsDNA committed B cell clones (CD20+) sensitive to B cell depletion, as well as from long‐lived (IgG) plasma cells (CD20−) 53. Autoantibody‐committed B cells are often preferentially removed by rituximab, as has also been shown in patients with RA 39, 54. A significant proportion of patients lost seropositivity to dsDNA at long‐term followup; however, there was little overall decrease in anti‐dsDNA antibodies in those patients with the highest baseline levels, suggesting the presence of a more entrenched autoreactive plasma cell pool.

The limitations of this study were that it was observational and from a single center, and that the data were not complete for all time points. However, clinical and laboratory results were available for the majority of patients at most recent followup (at least 54 of 57 patients); these results were complemented by prospective analysis of peripheral blood immunophenotyping of most patients who developed low serum IgM.

Our results showed that hypogammaglobulinemia after rituximab was largely restricted to the IgM class, and was associated with low baseline levels and a lower frequency of unswitched B cells. The development of low serum IgM hypogammaglobulinemia was also associated with sequential MMF. Monitoring of serum Ig levels is an important adjunct to the selection of concomitant/sequential immunosuppressants after B cell–depletion therapy. Reassuringly, low IgM after rituximab was not associated in our patients with increased risk of infections. Nonetheless, it would be prudent to continue surveillance of the patients for potential adverse events.

Taken together, the data presented provide new insights into the variability in biologic response, with rituximab providing useful information for the clinicians using rituximab for SLE.

## AUTHOR CONTRIBUTIONS

All authors were involved in drafting the article or revising it critically for important intellectual content, and all authors approved the final version to be submitted for publication. Dr. Cambridge had full access to all of the data in the study and takes responsibility for the integrity of the data and the accuracy of the data analysis.

### Study conception and design

Reddy, Leandro, Cambridge.

### Acquisition of data

Reddy, Martinez, Isenberg, Leandro.

### Analysis and interpretation of data

Reddy, Leandro, Cambridge.

## Supporting information

Supplementary Table 1: Patients with low serum IgM (<0.4g/L) post‐rituximab (n=12) at most recent follow up. Demographics, ethnic origin, clinical features and drug therapy are shown for individual patients.

Supplementary Table 2: Patients with serum IgM within the normal range (0.4‐2.3g/L) post‐rituximab (n=45) at most recent follow up. Demographics, ethnic origin, clinical features and drug therapy are shown for individual patients.

Supplementary Table 3: Demographics and laboratory parameters of patients studied prospectively for B‐cell phenotype (Figure 3C). Number of cycles of rituximab, co‐therapies and serology (C3 and anti‐dsDNA antibodies) of 9 SLE patients with immunoglobulin levels within the normal range after rituximab and in 8 patients with SLE who developed low serum IgM levels after rituximab.
