# Supplementary material for: Pragmatic Treatment of Patients With Systemic Lupus Erythematosus With Rituximab: Long‐Term Effects on Serum Immunoglobulins
Source: Arthritis Care Res (Hoboken). 2017 Apr 24;69(6):857–66. doi: 10.1002/acr.22993 (PMC5485064; doi:10.1002/acr.22993)
Supplement: Supplementary file 1 — Supplementary Table 1: Patients with low serum IgM (<0.4g/L) post‐rituximab (n=12) at most recent follow up. Demographics, ethnic origin, clinical features and drug therapy are shown for individual patients. [file ACR-69-857-s001.rtf]

Supplementary Table 1: Patients with low serum IgM (<0.4g/L) post-rituximab (n=12) at most recent follow up.  Demographics, ethnic origin, clinical features and drug therapy are shown for individual patients.

Patient number	Age	Clinical	RTX-	Treatment	Most recent
(years)	Ethnicity	manifestations	cycles	before RTX	treatment	Serology	
2	35	A	Non-renal	1	CS (high), AZA	CS (low), HCQ	ANA,Ro,RNP	
11	56	C	Non-renal	1	CS (low), AZA	Nil	RNP	
15	53	AC	LN (class 3)	1	CS (low)	CS (low)	RNP	
16	33	AC	LN (class 4)	2	CS (low)	CS (low), MMF (1.5g)	ENA-ve	
17	42	Ch	LN (class 5)	1	CS (low), MMF (3g), HCQ	CS (low), MMF (2g), Tacro	Ro,La,Sm,RNP	
22	51	AC	LN (class 3)	2	CS (low), AZA	MMF (2g)	ENA-ve	
30	55	C	LN (class 4)	1	CS (low), MMF (3g), HCQ	CS (low), HCQ, MMF (2g)	ENA-ve	
31	50	AC	Non-renal	1	CS (low), MTX	CS (low), Enbrel	Ro	
35	45	AC	LN (class 4)	2	CS (low), MMF (1.5g)	CS (low), HCQ	Sm	
43	53	AC	Non-renal	1	CS (low), HCQ	HCQ	Ro,La	
51	22	AC	LN (class 3)	1	HCQ, MMF (3g)	CS (low), HCQ, MMF (3g)	Ro,La	
53	46	C	Non-renal	2	CS (low), HCQ	Nil	Ro	
Abbreviations: A, Asian; AC, Afro-Caribbean; C, Caucasian;Ch, Chinese; LN, Lupus nephritis; Rituximab, RTX; dsDNA, (double stranded DNA); C3, complement component-3; HCQ, Hydroxychloroquine; CS, corticosteroids; CS (low), corticosteroids≤7.5mgs/day, CS (low), corticosteroids>7.5mgs/day AZT, azathioprine; MMF, mycophenolate mofetil; Tacro, Tacrolimus; ANA, anti-nuclear antibodies; RNP anti-ribo- nuclear proteins; ENA-ve, negative for anti-extractable nuclear antigens.
