# Supplementary material for: Pragmatic Treatment of Patients With Systemic Lupus Erythematosus With Rituximab: Long‐Term Effects on Serum Immunoglobulins
Source: Arthritis Care Res (Hoboken). 2017 Apr 24;69(6):857–66. doi: 10.1002/acr.22993 (PMC5485064; doi:10.1002/acr.22993)
Supplement: Supplementary file 2 — Supplementary Table 2: Patients with serum IgM within the normal range (0.4‐2.3g/L) post‐rituximab (n=45) at most recent follow up. Demographics, ethnic origin, clinical features and drug therapy are shown for individual patients. [file ACR-69-857-s002.rtf]

Supplementary Table 2: Patients with serum IgM within the normal range (0.4-2.3g/L) post-rituximab (n=45) at most recent follow up.  Demographics, ethnic origin, clinical features and drug therapy are shown for individual patients.


Patient number	Age (years)	Ethnicity	Clinical manifestations	RTX-
cycles	Treatment before RTX	Most recent treatment	Serology		
1	58	C	LN (class 4)	2	CS (high)	CS (low), HCQ	Ro, La		
3	28	AC	LN (class 3)	5	Aza, MMF, MTX, CYC	CS (low), HCQ	Ro,La		
4	34	A	LN (class 4)	1	Pred, HCQ, MMF (2g)	CS (low), HCQ, AZA	Ro, RNP		
5	33	AC	non-renal	1	CS (low), MTX	CS (low)	RNP		
6	29	A	LN (class 3)	5	CS (high), HCQ	CS (low), HCQ, Tacro, MMF (1.5g)	ENA-ve		
7	26	Ch	non-renal	1	CS (low), HCQ, AZA	CS (low), AZA	RNP		
8	31	Ch	non-renal	1	HCQ, AZA	HCQ, AZA	Sm		
9	43	C	non-renal	1	CS (low), AZA	HCQ, AZA	ENA-ve		
10	58	C	non-renal	1	CS (high)	CS (low)	Ro		
12	35	AC	LN (class 4)	1	HCQ, AZA	HCQ, AZA	RNP		
13	29	A	LN (class 4/5)	4	CS (low) MMF (2.5g)	CS (low), HCQ	Ro		
14	45	AC	LN (class 3)	1	CS (low) MMF (1.5g)	CS (low) MMF (0.5g), Tacr	Sm		
18	75	C	non-renal	3	CS (low), MTX	CS (low)	ENA-ve		
19	33	A	LN (class 3)	2	CS (low), HCQ, AZA	CS (low), HCQ, MMF (2g)	Ro, Sm, RNP		
20	28	C	LN (class 5)	1	CS (low), AZA	CS (low) Tacro	ENA-		
21	29	C	LN (class 4)	2	CS (low), HCQ, AZA	CS (low), HCQ, AZA	Ro, Sm, RNP		
23	54	A	LN (class 4)	1	CS (low) MMF (1.5g)	CS (low)	RNP		
24	29	AC	non-renal	1	CS (high)	CS (low)	ENA-ve		
25	30	C	LN (class 4)	1	CS (low), AZA	CS (low) MMF (1g), Tacro	ENA-ve		
26	30	Ch	non-renal	1	CS (low), MTX	IFX, MTX	ENA-ve		
27	29	A	LN (class 5)	1	CS (low) MMF (2g), HCQ	CS (low), HCQ	ENA-ve		
28	25	AC	LN (class 4)	2	CS (low), HCQ, AZA	CS (low), HCQ	Ro		
29	42	AC	LN (class 3)	1	CS (high)	CS (low)	Ro, Sm, RNP		
32	47	C	non-renal	1	CS (low), HCQ	CS (low), HCQ	Sm		
33	33	AC	LN (class 4)	3	CS (low), AZA	CS (low) MMF (2g)	Ro, RNP		
34	42	AC	non-renal	2	CS (high)	CS (low)	Ro, Sm, RNP		


36	40	C	non-renal	1	CS (low), HCQ	HCQ	ENA-ve	
37	30	AC	non-renal	2	HCQ	CS (low), HCQ	Ro	
38	27	AC	non-renal	1	CS (low), AZA	CS (low), HCQ	Ro	
39	21	A	LN (class 4)	2	CS (low), HCQ, AZA	HCQ	Sm	
40	19	AC	non-renal	2	CS (low), HCQ	CS (low), HCQ	RNP	
41	51	AC	LN (class 4)	1	CS (low)	CS (low), HCQ	La, Sm	
42	40	C	non-renal	2	CS (low), AZA	CS (low), HCQ	Ro, la	
44	22	C	non-renal	1	HCQ, MMF (1.5g)	HCQ	Ro, RNP	
45	45	AC	LN (class 4)	1	HCQ	nil	Ro La	
46	22	AC	LN (class 3)	4	CS (low), HCQ	CS (low) MMF (1.5g)	RNP	
47	47	C	LN (class 4)	2	CS (low), HCQ	CS (low), HCQ, MMF (2g)	Sm, RNP	
48	35	AC	non-renal	2	HCQ	CS (low)	ENA-	
49	35	AC	non-renal	2	HCQ	AZA	Ro, Sm, RNP	
50	29	C	non-renal	1	CS (low), HCQ, AZA	CS (low), AZA	Sm, RNP	
52	53	C	non-renal	2	HCQ, MTX	HCQ, MTX	Ro	
54	72	C	non-renal	2	AZA	CS (low)	RNP	
55	25	AC	LN (class 4/5)	1	CS (low) MMF (2g)	CS (low) MMF (1.5g)	Sm	
56	26	C	non-renal	2	CS (low) MMF (2g)	CS (low) MMF (1g)	Sm, RNP	
57	28		non-renal	2	CS (low) MMF (2g)	CS (low), AZA	ENA-ve	
Abbreviations: A, Asian; AC, Afro-Caribbean; C, Caucasian;Ch, Chinese; LN, Lupus nephritis; Rituximab, RTX; dsDNA, (double stranded DNA);
C3, complement component-3; HCQ, Hydroxychloroquine; CS, corticosteroids; CS (low), corticosteroids≤7.5mgs/day; CS (high), corticosteroids>7.5mgs/day; AZT, azathioprine; MMF, mycophenolate mofetil; Tacro, Tacrolimus; ANA, anti-nuclear antibodies; RNP anti-ribo- nuclear proteins; ENA-ve, negative for anti-extractable nuclear antigens.
