# Supplementary material for: Pragmatic Treatment of Patients With Systemic Lupus Erythematosus With Rituximab: Long‐Term Effects on Serum Immunoglobulins
Source: Arthritis Care Res (Hoboken). 2017 Apr 24;69(6):857–66. doi: 10.1002/acr.22993 (PMC5485064; doi:10.1002/acr.22993)
Supplement: Supplementary file 3 — Supplementary Table 3: Demographics and laboratory parameters of patients studied prospectively for B‐cell phenotype (Figure 3C). Number of cycles of rituximab, co‐therapies and serology (C3 and anti‐dsDNA antibodies) of 9 SLE patients with immunoglobulin levels within the normal range after rituximab and in 8 patients with SLE who developed low serum IgM levels after rituximab. [file ACR-69-857-s003.rtf]

Supplementary Table 3: Demographics and laboratory parameters of patients studied prospectively for B-cell phenotype (Figure 3C). Number of cycles of rituximab, co-therapies and serology (C3 and anti-dsDNA antibodies) of 9 SLE patients with immunoglobulin levels within the normal range after rituximab and in 8 patients with SLE who developed low serum IgM levels after rituximab.

IgA	IgG	IgM	CD19	RTX-	Post-RTX	Anti-dsDNA	C3	Co-therapies	
(g/L)	(g/L)	(g/L)	(%lymphocytes)	cumulative dose (g)	(months)	(IU/mL)	(g/L)		

Patients with immunoglobulin levels within the normal range after rituximab (n=9)

2.7	9.6	0.5	17.7	8	9	625	0.8	CS, HCQ	
2.7	12.7	0.5	2.7	8	36	27	1.1	CS	
3.0	10.9	1.4	9.1	2	9	115	1.2	CS, HCQ	
4.6	15.4	0.8	12.5	2	20	13	0.6	CS, HCQ	
3.2	13.9	0.7	1.3	2	15	58	1.1	AZT, HCQ	
4.6	7.0	1.1	9.5	4	9	17	0.7	HCQ, MMF	
4.7	12.2	0.8	6.6	2	72	153	0.8	CS, HCQ	
3.4	18.0	0.7	5.2	2	120	975	0.9	MTX, CS	
5.2	16.8	1.0	7.0	4	6	103	1.3	CS	

Patients who developed low serum IgM levels after rituximab (n=8)	
1.2	7.3	0.2	0.1	2	168	236	1.2	CS, HCQ	
2.9	4.3	0.1	2.0	8	24	37	0.9	CS, HCQ	
1.7	8.7	0.1	19.2	4	24	2	1.0	CS, HCQ	
2.9	11.7	0.4	3.7	2	60	254	0.9	AZT, CS	
1.2	14.4	0.3	7.0	4	24	246	1.0	CS, HCQ	
1.9	13.7	0.3	6.4	2	10	74	0.8	AZT, CS	
0.6	4.3	0.1	15.9	4	48	212	0.8	CS, MMF	
1.8	5.2	0.3	16.8	6	24	76	1.4	HCQ, MMF	
Abbreviations:	Rituximab,	RTX;	dsDNA  (double	stranded  DNA);	C3,  complement	component-3;	HCQ,	Hydroxychloroquine;  CS,	
corticosteroids; AZT, azathioprine; MMF, mycophenolate mofetil
